# Market Impact Analysis of Financial Literacy among A-Share Market Investors: An Agent-Based Model

**DOI:** 10.3390/e25121602

**Published:** 2023-11-29

**Authors:** Rongtian Zhou, Xiong Xiong, Bàrbara Llacay, Gilbert Peffer

**Affiliations:** 1 College of Management and Economics, Tianjin University, Weijin Road 92, Tianjin 300072, China; zhourongtian3315@163.com; 2 Laboratory of Computation and Analytics of Complex Management Systems (CACMS), Tianjin University, Weijin Road 92, Tianjin 300072, China; 3 Department of Business, Faculty of Economics and Business, University of Barcelona, Av. Diagonal 690, 08034 Barcelona, Spain; bllacay@ub.edu; 4 IAFI Research Group, Faculty of Economics and Business, Av. Diagonal 690, 08034 Barcelona, Spain; gilbert.peffer@gmail.com

**Keywords:** financial literacy, investor behaviour, agent-based model, A-share market

## Abstract

Financial literacy has become increasingly crucial in today’s complex financial markets. This paper explores the impact of financial literacy on the stock market by establishing an artificial financial market that aligns with the characteristics of the Chinese A-share market using agent-based modeling. The study incorporates financial literacy into investors’ mixed beliefs and simulates their behavior in the market. The results show that improving individual investors’ financial literacy can improve market quality and investor performance, as well as reduce the unequal distribution of wealth to some extent. However, the phenomenon of speculative trading and irrational behavior in the market can pose potential risks that require regulatory measures. Thus, policy recommendations to improve individual investors’ financial literacy and establish corresponding regulatory measures are proposed.

## 1. Introduction

With the development and complexity of financial markets, the importance of financial literacy is increasingly recognized. Financial literacy refers to an individual’s comprehensive understanding and application ability of financial knowledge, skills, and attitudes, including both personal financial management and the ability to use financial products and make investment decisions. An individual with high-level financial literacy can not only better manage their finances and investments, but also better adapt to, and respond to, changes and risks in financial markets [1].

Compared with the capital markets in developed countries, China’s capital market is relatively new, especially in the development of the stock market. In terms of financial literacy, there is a significant disparity between urban and rural areas in China, particularly in more remote rural areas. Simultaneously, Chinese investors may exhibit some unique investment habits, such as a greater inclination toward participating in the stock market and demonstrating relatively higher trading activity in stocks [2]. At the policy level, in recent years, the Chinese government has also started to address the issue of investor financial literacy and has repeatedly put forth policies related to investor financial education [3].

Existing research is trying to fully understand the complex relationship between financial literacy and the stock market. According to Gallego-Losada et al. (2022) [4], higher financial literacy is associated with a greater likelihood of engaging in financial information search and processing, and, thus, results in better performance. However, there has been a lack of research that can quantitatively and precisely measure the impact of financial literacy on investor behavior and the market in the stock market, especially for the Chinese A-share market where retail stock participation is higher. In-depth research on the impact of Chinese investors’ financial literacy on the A-share market becomes particularly meaningful. Therefore, in this paper, we integrate financial literacy with investor behavior by incorporating financial literacy into investors’ mixed beliefs and reflecting it in their ability to process financial news or events. A model based on agent-based modeling is established to measure the role of financial literacy in the stock market.

This paper aims to explore the role of financial literacy in the stock market through the agent-based modeling method and provide specific recommendations and plans. The main innovations of this study can be summarized in three aspects. Firstly, our study circumvents the complexity of measuring financial literacy and the potential for omitted variables, which is common in traditional empirical analysis methods. Instead, we innovatively approach the issue by focusing on the outcomes of financial literacy, successfully investigating how changes in financial literacy affect the market. Secondly, our study intricately classifies investors by capital scale during the measurement of financial literacy and provides policy recommendations for enhancing financial literacy tailored to specific investor categories. Lastly, our study fills a literature gap in the application of agent-based modeling to the topic of financial literacy. To be precise, we will first incorporate financial literacy into investors’ mixed beliefs, i.e., incorporate each investor’s financial literacy to reflect the proportions of their investment behavior attributable to fundamentalist, chartist and noise-induced components. Rather than delving into the intricate “black box” process of how financial literacy influences decision-making from the inception of investor financial literacy to decision provision, our model directly assesses the outcomes of financial literacy as the ability to make faster decisions and predict the future direction of stocks with greater accuracy. After that, we simulate the behavior of investors with different levels of financial literacy in the stock market and compare their investment performance and the resulting market impact. Using the agent-based modeling method, we will more accurately quantify the performance and market impact of investors with different levels of financial literacy and identify the factors that affect these performances and impacts. We will also determine what happens if the financial literacy of all investors or a particular group of investors is enhanced through certain measures. We hope that this research can contribute to improving the financial literacy of individuals and society, as well as promoting the stability and sustainable development of the financial market.

## 2. Literature Review

### 2.1. Financial Literacy

In recent years, financial literacy has become a popular research topic. Many scholars have extensively explored the concept, meaning, measurement methods, and influencing factors of financial literacy. In the stock market, many studies have also investigated the impact of financial literacy on investor behavior, market volatility, and the development of the stock market.

Several studies have examined the relationship between financial literacy and stock market participation. For example, in a study by Lusardi (2019) [5], individuals with higher levels of financial literacy were found to be more likely to participate in the stock market. This study also indicates that individuals with high financial literacy have greater opportunities to access information and can process information more efficiently. Cossa et al. (2022) [6] investigated the impact of financial literacy on individual financial well-being. They found that individuals with higher levels of financial literacy were more likely to hold stocks in their investment portfolio. Other studies have focused on the impact of financial literacy on stock market performance. Deuflhard et al. (2019) [7] examined the relationship between financial literacy and savings account returns. The authors found that individuals with higher levels of financial literacy tended to have higher savings account returns. Baker et al. (2019) [8] explored the relationship between financial literacy, demographic variables, and behavioral biases. The authors provided evidence to suggest that financial literacy can help individuals make better financial decisions.

In addition, several studies have examined the impact of financial literacy education on stock market outcomes. Compen et al. (2019) [9] examined the impact of financial literacy education on subsequent financial behavior and found that financial literacy education had a positive impact on stock market participation and investment behavior. Pettersson (2022) [10] found that financial literacy education had a positive impact on investment knowledge and behavior in the stock market. However, not all studies have found a positive relationship between financial literacy and stock market outcomes. Al-Bahrani et al. (2019) [11] found that financial literacy was not associated with stock market participation or investment behavior. Bottazzi and Lusardi (2021) [12] explored the relationship between financial literacy and savings behavior using data from the Programme for International Student Assessment (PISA). The result showed that financial literacy was not associated with stock market performance.

Furthermore, several studies have highlighted the importance of financial literacy in mitigating risk in the stock market. For instance, a study by Humaidi et al. (2020) [13] found that financial literacy was positively associated with risk management behavior in the stock market. Similarly, a study by Yang et al. (2018) [14] found that financial literacy was positively associated with the use of risk management strategies in the Chinese A-share market. Liao et al. (2017) [15] found that in terms of financial literacy’s impact on investor market participation and risk management, investors in the Chinese A-share market did not differ significantly from investors in other developed countries around the world.

In conclusion, previous research suggests that higher levels of financial literacy are associated with greater stock market participation, better investment behavior, higher stock market returns and better risk management. Financial literacy education may also have a positive impact on stock market outcomes.

Despite existing research attempts to understand the intricate relationship between financial literacy and the stock market, empirical studies often face challenges in capturing certain psychological or social factors due to the inherent complexity of financial literacy. Therefore, adopting an agent-based approach that is outcome-oriented from the perspective of financial literacy can yield more comprehensive evidence and conclusions, which is an area largely unexplored in the current literature.

### 2.2. Agent-Based Modeling

Agent-based modeling is a scientific research method based on computer models that simulate the behavior and interactions of autonomous agents in a complex system [16]. Agent-based models represent the system as a collection of individual agents, each with its own unique characteristics, rules, and behaviors. These agents interact with one another and with their environment, often resulting in emergent patterns and behaviors that can be difficult to predict using traditional mathematical models. Agent-based models can test and compare multiple variables in simulated financial markets, thereby more accurately predicting and evaluating changes and risks in financial markets. However, agent-based modeling may also have some limitations such as data requirements and validation challenges, as accurate agent-based modeling often demands detailed data on individual agents and their interactions, which may not always be readily available or feasible to collect, and researchers should ensure that the model’s behavior aligns with real-world observations and is a faithful representation of the system under study, which may be difficult.

The agent-based modeling method has been widely used to evaluate the effectiveness of investment portfolios, predict market volatility, and other aspects. Yang et al. (2022) [17] discussed the use of the method in studying market microstructure, financial crises, and market regulation. Dehkordi et al. (2023) [18] provided a comprehensive overview of the use of agent-based modeling in finance, including its strengths and weaknesses. They argued that the method can provide valuable insights into market behavior, but that care must be taken in constructing the models to ensure they accurately reflect the real-world dynamics of financial markets. Axtell and Farmer (2020) [19] reviewed the use of the method in studying market stability, asset pricing, and other aspects of finance. They argued that agent-based modeling has the potential to transform our understanding of financial markets and improve our ability to predict and prevent financial crises.

In empirical study, the influence of financial literacy on stock market participation and financial behavior is well established. However, in this work, the agent-based modeling method has unique advantages. First, it can consider investors with different levels of financial literacy in the experimental environment, avoiding interference factors that are difficult to control in the actual market. However, in empirical research, there is a possibility of omitted variables or a lack of certain unobservable changes in investor sentiment or psychological aspects [20]. Second, the agent-based modeling method can quickly generate a large amount of data, thereby improving the accuracy and credibility of the research [21]. Finally, through the analysis of the experimental results, we can determine what will happen if we implement certain measures to enhance the financial literacy of all investors or a particular group of investors, such as strengthening information disclosure or providing more investor education. These measures require significant costs to implement. In particular, investor education is a long-term and resource-intensive undertaking, and its effectiveness is uncertain. Empirical research can only analyze outcomes that have already occurred, while agent-based models have a forward-looking nature, which can reduce the trial-and-error costs of policies. Therefore, through agent-based modeling, we can simulate their effects and provide evidence-based policy recommendations.

## 3. Model Description

### 3.1. Market Design

In this section, we describe an artificial stock market designed to emulate the characteristics of the Chinese A-share market. The synthetic market comprised four segments: Shanghai main-board (SHM Market), Shenzhen main-board (SZM Market), Second-board (SB Market), and Sci-Tech Innovation board (STAR Market). Real A-share market data were employed to fine-tune the features of stock prices, equity, and volatility within these four sectors (refer to Appendix A for calibration results).

To align with the real market’s attributes, we created a simplified limit order book market model that adhered to specific traits of the Chinese stock market, including:

(1) Each stock possessing an independent order book, resulting in four order books within the artificial stock market;
(2) The minimum quotation unit being RMB 0.01;
(3) Emptying the order book at the close of each trading day;
(4) The absence of a call auction—the opening price of each stock on a trading day was the closing price of the previous day;
(5) Each simulation cycle corresponded to 1 min in the real market, mirroring the 4 h trading time of each trading day in the Chinese stock market. In other words, 240 simulation cycles represented one trading day;
(6) Setting differential price limits for distinct sectors: a 10% price limit for the main board (SHM Market and SZM Market), and a 20% limit for the SB Market and STAR Market;
(7) According to the 2022 Shanghai Stock Exchange Statistics, the monthly proportion of short selling transactions in the A-share market was less than 1% for nearly 80% of the time. Therefore, for the sake of model simplification, short selling was not allowed. (The characteristics and trading rules of the artificial stock market model constructed in this study were derived from the real rules outlined in the *Trading Rules of Shenzhen Stock Exchange*.)

### 3.2. Trader Types and Structure

There were 5000 traders in the model. To examine the impact of financial literacy changes on different traders, we classified all individual accounts into five categories based on account-level transaction data: (1) Retail: less than RMB 100,000; (2) Sinvestor: between RMB 100,000 and 500,000; (3) Minvestor: between RMB 500,000 and 5 million; (4) Linvestor: between RMB 5 million and 10 million; and (5) XLinvestor: larger than RMB 10 million. We also included institution accounts as a separate group, which included mutual funds, insurance companies, security firms, and pension funds (the investor structure data of Shanghai main-board (SHM Market) and Technology Innovation Board (STAR Market) were drawn from the 2019 Shanghai Stock Exchange Statistics. The investor structure data of Shenzhen main-board (SZM Market) and Second-board (SB Market) were derived from the *2019 Investor Structure and Behavior Analysis Report of Shenzhen Stock Exchange*). The specific number and wealth distribution of the five categories of investors can be found in Section 4.1, where the parameters of the model are set.

Next, we defined the portfolio wealth for each agent. Upon entering the market initially, traders received an allocation of both stock and cash. The initial stock position $S_{0}^{i,j}$ for agent i was:

(1) $$S_{0}^{i,j}=S_{Mean}^{j}\varphi$$

where $S_{Mean}^{j}$ was the position of each trader equally allocated to stock j, and φ followed a uniform distribution between 0.01 and 0.99.

The assumed initial cash position $\alpha_{0}^{i}\;$ for agent i was:

(2) $$\alpha_{0}^{i}=\sum_{j=SHM,SZM,SB,STAR}p_{0}^{j}S_{0}^{i,j}$$

where $p_{0}^{j}\;$ was the initial price of stock j. The optimal composition of the agent’s portfolio was determined in the usual way by trading-off expected return against expected risk. However, the agents were not allowed to engage in short selling. Specifically, when the total wealth of a trader was negative, we considered the trader to be bankrupt. At this time, a new trader would enter the market to ensure the normal operation of the agent-based model (Based on simulation data, during normal market operations, the percentage of such traders was minimal, and the augmented wealth of investors had an insignificant impact on the market. In the event of a market crash, the proportion of new traders would expand. However, the primary market driver did not lie in augmenting traders' wealth, thereby resulting in a negligible impact on the model.)

### 3.3. Traders Financial Literacy and Price Expectations

Financial literacy is a complex factor encompassing the information processing ability mentioned, along with various other socioeconomic or psychological abilities. It is challenging to articulate this comprehensively in both empirical and experimental research. Fortunately, in an agent-based model, we can measure an individual’s level of financial literacy by their precise control over the outcome of a particular event. For instance, in the case of negative news, investors with high financial literacy are more likely to quickly acquire information, synthesize various factors such as market sentiment, socioeconomic conditions, and personal considerations, and efficiently draw conclusions about the decline in stock prices. They can also make more accurate predictions about the extent of the decline. On the other hand, investors with low financial literacy may acquire information more slowly and arrive at conclusions with greater deviations. The process from the appearance of information to drawing conclusions involves a series of “black box” operations, including information acquisition and internal cognitive processes, which are not observable. However, in an agent-based model, we can infer from the results: investors with high financial literacy will exhibit differences in the speed and accuracy of their reactions to market information, compared with those with low financial literacy.

In accordance with Chiarella et al. (2017) [22], the demand for the risky asset by each trader was assumed to comprise three components: a fundamentalist component, a chartist component, and a noise-induced component. However, rather than simply using statistical data as parameters, we incorporated each investor’s financial literacy to reflect the proportions of their investment behavior attributable to fundamentalist, chartist and noise-induced components. Furthermore, the fundamental value of stocks in the market was not transparent or fixed, but rather varied among individuals and changed with time and various events. The level of financial literacy reflects investors’ abilities to obtain information and process events, thereby affecting their estimation of the fundamental value of stocks and their sensitivity to changes in fundamental value.

At any time, t, a trader was chosen to enter the market. The chosen agent, i, formed an expectation about the stock return, $r_{t+\tau }^{i,j}$, where $\tau_{i}$ represents the agent’s time horizon. Agents utilized a blend of fundamental value and chartist rules to shape expectations regarding stock returns, resulting in:

(3) $$r_{t+\tau }^{i,j}=x^{i}r_{c}^{i,j}+y^{i}r_{f}^{i,j}+z^{i}\varepsilon$$

where the quantities $x^{i}$ and $y^{i}$ represent the weights given to the chartist and fundamentalist components, respectively. For normalization purposes, we assumed that $x^{i}+y^{i}+z^{i}$ = 1.

We assumed that $x^{i}=\frac{\beta }{e^{L_{i}}}$ and $z^{i}=(1-\beta )\left(1-L_{i}\right),$ where $L_{i}$ is the financial literacy of agent i, and β represents the conversion strength of investor financial literacy. Barber and Odean (2000) [23] indicated that investors with a high level of financial literacy are more inclined to make investment decisions by thoroughly analyzing fundamental factors such as a company’s financial statements and industry prospects. They are also more likely to opt for long-term stock holdings rather than frequent trading. The study also indicated that 20% of trades are noise trading and are hazardous. Therefore, the β was set as 80% in our model. Equation (3) also means that if an investor is completely lacking in financial literacy, i.e., $L_{i}=0$ (which will not happen in most cases), they will be completely unable to access useful financial news or events, or obtain price information from such news or events. Therefore, they would have no choice but to be a pure momentum-based trader, relying solely on the historical price trend to make their stock price predictions with a maximum proportion of noise-induced component of 0.2. In another extreme case, i.e., when $L_{i}=1$, the investor would have full information of the market and would be able to immediately perceive the fundamental changes in the value of a stock and accurately determine its value. In this case, the trader would still have speculative motives ($x^{i}>0$), which means they would also engage in momentum trading and profit from price fluctuations. $x^{i}=\frac{\beta }{e^{L_{i}}}$ also indicates that the higher the financial literacy of agent i, $L_{i}$, the smaller the proportion of chartist components the agent will have, while there is a diminishing marginal benefit to improving financial literacy. The variable ε, featuring a zero mean and variance $\sigma_{\varepsilon }$ to agent’s expectations, signifies the noisy beliefs of investors.

$r_{c}^{i,j}\;$ indicates the anticipated future trend of the chartist component derived from observations of spot returns over the last $\tau_{i}\;$ time steps. In other words,

(4) $$r_{c}^{i,j}=ln\frac{{\bar{p}}_{\tau_{i/4}}^{j}}{{\bar{p}}_{\tau_{i}}^{j}}$$

where ${\bar{p}}_{\tau_{i/4}}^{j}$ is the short-term average price of stock j, and ${\bar{p}}_{\tau_{i}}^{j}$ is the long-term average price of stock j.

$r_{f}^{i,j}$ represents the trader’s predicted return on stock *j* based on fundamental beliefs, that is,

(5) $$r_{f}^{i,j}=\frac{1}{\tau_{i}}(ln\frac{{\hat{f}}_{t}^{j}}{p_{t}^{j}})$$

where $p_{t}^{j}$ is the price of stock j at time t. The variable $\tau_{i}\;$ represents the time scale over which the fundamentalist component calculates the mean reversion of the price to the fundamental. ${\hat{f}}_{t}^{i,j}$ is the fundamental value of stock j predicted by agent i, that is,

(6) $${\hat{f}}_{t}^{i,j}=L_{i}\cdot f_{t}^{j}+\left(1-L_{i}\right)\cdot {\bar{p}}_{\tau_{i}}^{j}$$

where $f_{t}^{j}\;$ is the real fundamental value of stock j at time t. Equation (6) means that the higher the financial literacy, the less the agents will be influenced by historical price trends, and their predictions will be closer to the real fundamental value of stocks.

It is common to assume that the time horizon of an agent depends on its characteristics. Fundamentalist strategies are typically given much greater weight by long term institutional investors who have longer time horizons, whilst day traders have shorter time horizons and give more weight to chartist rules. Hence, we chose the time horizon $\tau^{i}$ of each agent according to:

(7) $$\tau^{i}=\tau^{*}\frac{1+y^{i}}{1+x^{i}}$$

$$\tau^{*}=5day=1200t$$

We assumed that each trader arrived to the market according to a Poisson process with parameter $\lambda^{i}=\omega /\tau^{i}$ and traded continuously.

The future price, $p_{f,t}^{i,j}\;$, expected at time t by agent i was given by:

(8) $$p_{f,t}^{i,j}=p_{t}^{j}e^{r_{t+\tau }^{i,j}\tau^{i}}$$

### 3.4. Order Submission Rules

Traders trade only when their expected order profit from trading is high enough to offset the transaction cost. In a dynamic equilibrium model of an order driven market with asymmetric information, Foucault et al. (2016) [24] showed that informed traders submit both market orders and limit orders, depending on whether their information advantage is above or below a cutoff value. Gil-Bazo and Ruiz-Verdú (2009) [25] introduced similar order submission rules, being that traders submit market orders when the price deviations from their forecasting fundamental value are large, and limit orders when the deviations are small. Depending on traders’ forecasting and order book states, traders submit either limit or market orders. We introduced similar order submission rules. When trader i arrived in the market at time t, within the time period t, they assessed their expected price $p_{f,t}^{i,j}$ against the current best bid $b_{t}^{j}$ and best ask $a_{t}^{j}$ factoring in the transaction cost $u=1\%p_{t}^{m,j}$, where $p_{t}^{m,j}$ was the midpoint of the optimal bid and ask quotes. Depending on the current order book, following Sornette and Zhou (2006) [26], we considered four scenarios that aligned with the characteristics of the order book in the Chinese A-share market, summarized in Table 1. In the first scenario, where there was at least one ask and one bid in the current limit order book, the trader placed a market order to buy if their expected price $p_{f,t}^{i,j}$ was above the sum of the best ask $a_{t}^{j}$ and the transaction cost u, i.e., $p_{f,t}^{i,j}-u>a_{t}^{j}$. Conversely, if their expected price $p_{f,t}^{i,j}$ was below the best bid $b_{t}^{j}$ minus the transaction cost u, i.e., $p_{f,t}^{i,j}+u<b_{t}^{j}$, they placed a market order to sell. In case of $\left[p_{f,t}^{i,j}\geq p_{t}^{m,j}]\&[a_{t}^{j}+u\geq p_{f,t}^{i,j}\geq b_{t}^{j}-u\right]$, the trader submitted a limit buy order. For $p_{f,t}^{i,j}<p_{t}^{m,j}]\&[a_{t}^{j}+u\geq p_{f,t}^{i,j}\geq b_{t}^{j}-u$, they submitted a limit sell order, depending on whether their expected price $p_{f,t}^{i,j}$ was above or below the current $p_{t}^{m,j}$. The rules for order submission in the other three cases were defined in a similar manner.

## 4. Experiment

Utilizing the agent-based model and the previously introduced order mechanism for traders, we assessed the effectiveness and repercussions of alterations in financial literacy.

To demonstrate the differences in financial literacy among investors in information processing, we simulated a real-world scenario in which an unexpected event occurred. This meant that the fundamental value of a stock would suddenly change (either increase or decrease) at some point due to the occurrence of the event. Financial literacy determines an investor’s ability to process information, which, in turn, determines their ability and efficiency in obtaining the new fundamental value of the stock. Investors rely on their own judgment to assess the fundamental value of the stock, but this judgment is also influenced by recent price changes of the stock. There is a significant difference in financial literacy between individual investors and institutional investors. According to Meng et al. (2020) [27], we set the financial literacy ($L_{i}$) of individual investors to follow a normal distribution with a mean of 0.22 and a standard deviation of 0.075. The financial literacy ($L_{i}$) of institutional investors was set as a normal distribution with a mean of 0.48 and a standard deviation of 0.055.

In this section, we first examine the degree to which financial literacy affects investors, that is to say, whether having financial literacy causes investors to perform better in trading during unexpected events. Then we consider a model with various financial literacy levels among investors and examine the effect of changes in financial literacy for different groups of investors. For the purpose of controlling variables, during the experimental process, we fixed the random factors of the experiment. In other words, in the paired comparative experiments where the two groups corresponded one-to-one, we controlled all other factors, including market conditions, timing of investors entering the market, occurrence of unforeseen events, and their impact. The only difference was the varying levels of financial literacy among investors, ensuring that any observed variations in the final results were solely due to changes in investors’ financial literacy.

### 4.1. Benchmark Model

For the benchmark model (BM), we assumed that the fundamentalism, chartism and noise trading of investors were unrelated to financial literacy, and investors could only derive judgments about future stock price movements through analysis of historical price trends. The purpose of setting up this benchmark model was to compare it against other scenarios, thus, demonstrating that financial literacy does have an impact on market quality and investor performance.

The number of traders was assumed as 5000, i.e., N=5000. We calibrated the trade structure and wealth levels of different types of traders based on the data of 2019 Shanghai Stock Exchange Statistics and the *2019 Trade Structure and Behavior Analysis Report of Shenzhen Stock Exchange*, as shown in Table 2. Based on Goettler et al. (2009) [28] and Karpe et al. (2020) [29], we assumed that each trader entered the market according to a Poisson process with a parameter λ and engaged in continuous trading. In the model, one day corresponded to 240 simulation cycles, denoted as D=240.

Using the specified parameter values, we conducted 30 simulations with distinct random seeds to ensure statistical significance. As traders require ample time to learn optimal forecasting rules, each simulation ran for 60,000 periods. The analysis focused on the last *T* = 12,000 periods, equivalent to approximately 200 h. (According to the SFI-ASM [30], in general, 400 generations are long enough to allow a GA with a classifier system to evolve to an optimal level, so, 48,000 periods were needed in our model.) To assess the impact of learning and its interaction with information lag, fundamental value volatility, and the number of informed traders, we examined four scenarios, as outlined in Table 3.

### 4.2. Scenarios A and B: Financial Literacy for All Investors and Improved Financial Literacy for Individual Investors

In scenarios A and B, we assumed that the degrees of fundamentalism, chartism and noise trading were determined by the financial literacy of both institutional investors and individual investors. According to Meng et al. (2020) [27], in scenario A, we set the financial literacy ($L_{i}$) of institutional and individual investors to follow normal distributions of $N\left(0.48,0.055^{2}\right)$ and $\left(0.22,0.075^{2}\right)$, respectively. In scenario B, we simulated a scenario of enhanced market disclosure and investor education, where we raised the financial literacy of individual investors to the same level as institutional investors, i.e., the financial literacy ($L_{i}$) of all investors followed a normal distribution of $N\left(0.48,0.055^{2}\right)$. In comparing scenario A with the BM model, our goal was to illustrate the relationship between financial literacy and mixed beliefs, and to assess the extent to which introducing financial literacy to investors influences their returns and the overall market quality. In the comparison of scenario B with the BM model, our aim was to explore the effects of initiatives aimed at improving market disclosure and enhancing financial literacy education for individual investors on investor performance and overall market quality.

### 4.3. Scenarios C and D: Financial Literacy Only for Institutional Investors or Individual Investors

In scenarios C and D, we assumed that the degrees of fundamentalism, chartism and noise trading were determined by the financial literacy of institutional investors or individual investors separately. Similarly, in scenario C, we set the financial literacy ($L_{i}$) of institutional investors to follow a normal distribution with a mean of 0.48 and a standard deviation of 0.055, which was denoted as $N(0.48,0.055^{2})$. In scenario D, the financial literacy ($L_{i}$) of individual investors was set to follow a normal distribution, $N(0.22,0.075^{2})$. By comparing scenarios C and D with each other and with the BM model, we aimed to further investigate the role of financial literacy in the stock market. By separately introducing financial literacy to institutional (scenario C) or individual investors (scenarios D), we studied the degree and differences in the impact on investor returns and overall market quality, in order to determine whether improving financial literacy was always better for all groups.

## 5. Results

### 5.1. Scenarios A and B, and BM Model

In this work, we used the volatility of the transaction price and the volatility of the mid-point of bid and ask prices to measure the volatility of the market. As illustrated in Table 4, when financial literacy was introduced to all investors in the market (BM and scenario A), the volatility of overall market decreased, with small-cap (STAR Market) stocks experiencing a more significant decline in volatility compared with mid-cap stocks (SZM Market and SB Market), while large-cap stocks (SHM Market) had almost no change in volatility. The reason for this may be that as investors gain financial literacy, their speculative interest in volatile mid- and small-cap stocks decreases, leading to a reduction in volatility in these stocks. Therefore, the problem of high volatility in mid- and small-cap stocks has been somewhat alleviated. When we improved the financial literacy of individual investors through certain measures (scenarios A and B), the overall market volatility did not show a significant difference compared with before the improvement. A reason for this might be that individual investors, compared with institutional investors, have a smaller amount of capital and, therefore, their impact on the market is not significant enough to cause a noticeable difference in overall volatility.

Bid–ask spread and trading volume are used to measure the liquidity of the market. As shown in Table 5, the introduction of financial literacy (BM and scenario A) did not lead to a significant change in the overall bid–ask spread of the market. However, there was a decrease in trading volume, which may be attributed to the fact that investors with improved financial literacy engage in more rational trading behavior; as a result, short-term speculative trading decreases while long-term investment trading increases. On the other hand, when individual investors’ financial literacy was improved (scenario A and B), there was no significant difference observed in the overall market liquidity and trading volume.

We used the MAE and MRE indicators to measure the pricing efficiency of the market, with smaller values indicating higher pricing efficiency. MAE and MRE, respectively, represent the absolute deviation and relative deviation of market price from asset fundamental value. The formulas used were:

(9) $$MAE=\frac{1}{T}\sum_{t=1}^{T}\left|p_{t}-f_{t}\right|$$

(10) $$MRE=\frac{1}{T}\sum_{t=1}^{T}\frac{\left|p_{t}-f_{t}\right|}{f_{t}}$$

where $p_{t}$ represents the asset price at time t, and $f_{t}$ denotes the asset fundamental value at time t. The MAE and MRE values are the averages derived from 30 experimental trials. Paired *t*-tests on MAE and MRE is also conducted, with the resulting p-values presented in parentheses. Significant p-values are bolded.

As shown in Table 6, with the introduction of financial literacy (BM and scenario A), when the overall market p-value was less than 0.01, the overall market pricing efficiency significantly improved. Examining individual sectors, both MAE and MRE *p*-values for the SB Market were greater than 0.05. With the exception of the MAE for the STAR Market, which had a *p*-value less than 0.05, the *p*-values for other sectors were all less than 0.01. This suggests a significant enhancement in pricing efficiency for large-cap stocks, a weaker but still significant improvement for small-cap stocks, and no significant impact on mid-cap stocks, from the introduction of financial literacy. When enhancing the financial literacy of individual investors separately (scenarios A and B), the overall market pricing efficiency further significantly improved. The improvement in pricing efficiency for large-cap stocks exceeded that for small-cap stocks, while mid-cap stocks still experienced no significant impact.

At the investor level, we used an annualized return rate and maximum drawdown to evaluate investment performance. As shown in Table 7, with the introduction of financial literacy (BM and scenario A), the maximum drawdown of different groups of investors significantly decreased, indicating that financial literacy can significantly improve investors’ risk management capabilities. In terms of returns, institutional investors’ returns increased significantly, while the returns of XLinvestor and Linvestor also increased, but to a lesser extent than institutional investors. However, investors with smaller fund sizes showed a significant decrease in returns. The reason may be that institutional investors have higher financial literacy, which allows them to detect market changes earlier and make timely adjustments. Investors with larger funds can diversify their positions to reduce investment risks and make profits, while investors with smaller funds can only passively bear the risks and losses brought by price fluctuations. When personal investors’ financial literacy was improved (scenarios A and B), institutions’ returns declined, and the drawdown rate significantly increased while the overall returns of personal investors increased. The drawdown rate of XLinvestor and Linvestor also significantly decreased, and the drawdown rate of retailers also decreased. The results showed that improving individual investors’ financial literacy can alleviate the phenomenon of information asymmetry in the market, thereby alleviating the unequal distribution of wealth to some extent.

### 5.2. Scenarios C and D, and BM Model

By comparing scenarios C and D with each other and with the BM model, we aimed to further investigate the role of financial literacy in the stock market.

As shown in Table 8, when only institutions possessed financial literacy (BM and scenario C), there was a slight decrease in market volatility. In contrast, when only individual investors possessed financial literacy (BM and scenario D), there was a slight increase in market volatility. In addition, the volatility of large-cap stocks increased more than that of mid-cap stocks. This could be attributed to the fact that when a minority of market participants (individual investors) become more knowledgeable, they are more likely to actively engage in speculation alongside the mainstream market funds (institutions). Consequently, this exacerbates the increase in market volatility.

As shown in Table 9, bid–ask spreads of the market decreased in both cases, indicating that financial literacy had a positive impact on market liquidity. However, regarding trading volume, when only institutions possessed financial literacy (BM and scenario C), there was a slight decrease in market volume, while when only individual investors possessed financial literacy (BM and scenario D), there was a slight increase in market trading volume. Similar to the increase in market volatility, this may be due to the fact that when a minority of market participants (individual investors) become more knowledgeable, they are more likely to actively engage in speculation alongside the mainstream market funds (institutions). As a result, this contributes to some extent to the increase in market trading volume. At the sector level, when only institutions possessed financial literacy, the bid–ask spread for large-cap and small-cap stocks narrowed, while the bid–ask spread for mid-cap stocks widened. This may be attributed to two factors: institutional investors paying more attention to the long-term investment value of large-cap stocks after acquiring financial literacy, while also seeking short-term profit opportunities in high-volatility small-cap stocks. Due to this attention-grabbing effect, mid-cap stocks were consequently overlooked, resulting in reduced liquidity.

As indicated in Table 10, by comparing scenarios C and D with each other and with the BM model, all *p*-values were greater than 0.05. Therefore, the singular factor of institutional or individual investor financial literacy did not have a significant impact on market pricing efficiency. This result also confirmed the phenomenon in the A-share market where individual investors tend to participate actively in stock trading, and the market is not entirely dominated by institutional investors.

Regarding investor performance, as can be seen in Table 11, it was observed that when only institutions possessed financial literacy (BM and scenario C), except for the Retailer group, all other investor groups experienced an increase in returns, while the maximum drawdown for all investors decreased. In contrast, when only individual investors possessed financial literacy (BM and scenario D), only the Sinvestor and Retailer groups achieved positive returns, while other investor groups experienced varying degrees of decreases in returns. Additionally, the drawdown rate for Institution and XLinvestor significantly increased, while the drawdown rate for Sinvestor and Retailer slightly decreased. Thus, it can be inferred that increasing the financial literacy of mainstream market funds can improve the overall risk management capability of the market, although it may result in losses for Retailers. Conversely, increasing the financial literacy of only a small portion of the funds may provide gains for individual investors, but it may also increase the overall market risk.

## 6. Conclusions

In this paper, we created an artificial financial market that mirrors the characteristics of the Chinese stock market. Agent-based methods were used to study the impact of investor financial literacy on market quality and investor performance.

The results of the study indicated that from the perspective of market quality, the introduction of financial literacy can effectively reduce market volatility, especially for small-cap stocks. Although it may decrease trading volume, it can significantly improve market pricing efficiency. In terms of investor performance, the introduction of financial literacy can significantly increase investors’ risk management ability and improve institutions’ return on investment, while small individual investors’ return on investment may decrease. Meanwhile, measures such as strengthening information disclosure or investor education, which improve individual investors’ financial literacy, can significantly improve individual investors’ returns. Therefore, improving individual investors’ financial literacy can alleviate the phenomenon of information asymmetry in the market, thereby alleviating the unequal distribution of wealth to some extent. However, the experimental results also indicated that when the mainstream capital in the market mainly engages in speculative trading, more “smart” individual investors tend to adopt a follow-up strategy, which may cause an increase in market volatility and investor risk.

Based on the summary, several policy recommendations can be proposed. First, due to the fact that individual investors constitute the vast majority in terms of quantity and are more active in trading in the Chinese A-share market, it is advisable to implement policy measures aimed at enhancing the financial literacy of individual investors, such as strengthening information disclosure and investor education. This can effectively alleviate the phenomenon of information asymmetry in the market, thereby reducing the unequal distribution of wealth to some extent. Second, it is necessary to be vigilant about speculative trading by institutional investors in the A-share market. According to the experimental results, individual investors with higher financial literacy in the A-share market tend to adopt a strategy of following the trading behavior of institutional investors. Therefore, regulating the behavior of institutional investors becomes particularly important. Regulatory authorities in the A-share market should establish stricter regulatory measures for institutional investors to prevent excessive speculation and ensure the stable operation of the market.

The limitations of this study lie in two aspects: firstly, the current model is only applicable to the Chinese A-share market, and secondly, the impact of unforeseen events is only manifested as a disruption to the fundamental value of stocks. Therefore, future research could be divided into two main directions. Firstly, the agent-based model can be extended to the financial literacy applications in other global markets. Secondly, there is a need to refine the types of unforeseen events and their impacts, in order to investigate how financial literacy plays a role in market quality and investor performance when dealing with different events.

## Figures and Tables

**Table 1:** Agent order submission rules.

Scenario	Order Type	Order Price
**Scenario 1: At least one ask and one bid in order book.**		
$p_{f,t}^{i,j}-u>a_{t}^{j}$	Market buy	$p_{mb}^{i,j}<p_{f,t}^{i,j}-u$
$\left[p_{f,t}^{i,j}\geq p_{t}^{m,j}]\&[a_{t}^{j}+u\geq p_{f,t}^{i,j}\geq b_{t}^{j}-u\right]$	Limit buy	$p_{lb}^{i,j}=p_{f,t}^{i,j}-u$
$\left[p_{f,t}^{i,j}<p_{t}^{m,j}]\&[a_{t}^{j}+u\geq p_{f,t}^{i,j}\geq b_{t}^{j}-u\right]$	Limit sell	$p_{ls}^{i,j}=p_{f,t}^{i,j}+u$
$p_{f,t}^{i,j}+u<b_{t}^{j}$	Market sell	$p_{ms}^{i,j}>p_{f,t}^{i,j}+u$
**Scenario 2: No bids in order book.**		
$p_{f,t}^{i,j}-u>a_{t}^{j}$	Market buy	$p_{mb}^{i,j}<p_{f,t}^{i,j}-u$
$p_{f,t}^{i,j}-u\leq a_{t}^{j}$	Limit buy	$p_{lb}^{i,j}=p_{f,t}^{i,j}-u$
**Scenario 3: No asks in order book.**		
$p_{f,t}^{i,j}+u<b_{t}^{j}$	Market sell	$p_{ms}^{i,j}>p_{f,t}^{i,j}+u$
$p_{f,t}^{i,j}+u\geq b_{t}^{j}$	Limit sell	$p_{ls}^{i,j}=p_{f,t}^{i,j}+u$
**Scenario 4: No bids or asks in order book.**		
50%	Limit buy	$p_{lb}^{i,j}=p_{f,t}^{i,j}-u$
50%	Limit sell	$p_{ls}^{i,j}=p_{f,t}^{i,j}+u$

**Table 2:** Calibrating the trade structure and wealth levels of different types of traders.

Panel A: Shanghai main-board			
Trader names	Trader types	Number of Traders	Proportion of wealth
Retailer	Less than RMB 100,000	2700	1.25%
Sinvestor	Between RMB 100,000 and 500,000	1500	3.50%
Minvestor	Between RMB 500,000 and 1,000,000	680	5.46%
Linvestor	Between RMB 1,000,000 and 5,000,000	85	2.78%
XLinvestor	More than RMB 5,000,000	25	6.63%
Institution	Institutional investor	10	80.38%
**Panel B: Shenzhen main-board**			
Trader names	Trader types	Number of Traders	Proportion of wealth
Retailer	Less than RMB 100,000	2700	1.59%
Sinvestor	Between RMB 100,000 and 500,000	1500	5.37%
Minvestor	Between RMB 500,000 and 1,000,000	680	9.52%
Linvestor	Between RMB 1,000,000 and 5,000,000	85	4.97%
XLinvestor	More than RMB 5,000,000	25	22.11%
Institution	Institutional investor	10	56.44%
**Panel C: Second-board**			
Trader names	Trader types	Number of Traders	Proportion of wealth
Retailer	Less than RMB 100,000	2700	1.16%
Sinvestor	Between RMB 100,000 and 500,000	1500	5.72%
Minvestor	Between RMB 500,000 and 1,000,000	680	11.24%
Linvestor	Between RMB 1,000,000 and 5,000,000	85	5.99%
XLinvestor	More than RMB 5,000,000	25	35.07%
Institution	Institutional investor	10	40.82%
**Panel D: Sci-Tech Innovation board**			
Trader names	Trader types	Number of Traders	Proportion of wealth
Retailer	Less than RMB 100,000	2700	0%
Sinvestor	Between RMB 100,000 and 500,000	1500	0%
Minvestor	Between RMB 500,000 and 1,000,000	680	12.07%
Linvestor	Between RMB 1,000,000 and 5,000,000	85	6.43%
XLinvestor	More than RMB 5,000,000	25	37.66%
Institution	Institutional investor	10	43.84%

**Table 3:** Detail of each scenario.

Financial Literacy	Institutional Investors	Individual Investors
Scenario A	$\sim \;N(0.48,0.055^{2})$	$\sim \;N(0.22,0.075^{2})$
Scenario B	$\sim \;N(0.48,0.055^{2})$	$\sim \;N(0.48,0.055^{2})$
Scenario C	$\sim \;N(0.48,0.055^{2})$	0
Scenario D	0	$\sim \;N(0.22,0.075^{2})$

**Table 4:** Market volatility results for scenarios A and B, and BM model.

Volatility of Transaction Price	SHM Market	SZM Market	SB Market	STAR Market	Mean
Benchmark Model (BM)	66.0	73.6	77.0	84.9	75.4
Scenario A	67.0	70.8	73.7	76.4	72.0
Scenario B	66.3	71.1	75.6	75.8	72.2
Relative deviation of BM-A	1.60%	−3.77%	−4.34%	−10.03%	−4.50%
Relative deviation of A-B	−1.06%	0.47%	2.53%	−0.81%	0.30%
**Volatility of mid-point of bid and ask prices**	**SHM Market**	**SZM Market**	**SB Market**	**STAR Market**	**Mean**
Benchmark Model (BM)	57.4	67.2	70.6	92.0	71.8
Scenario A	57.4	63.3	67.0	80.0	66.9
Scenario B	56.4	63.5	69.4	78.0	66.8
Relative deviation of BM-A	−0.10%	−5.82%	−5.17%	−13.07%	−6.84%
Relative deviation of A-B	−1.74%	0.41%	3.67%	−2.53%	−0.11%

**Table 5:** Market liquidity results for scenarios A and B, and BM model.

Bid–Ask Spread	SHM Market	SZM Market	SB Market	STAR Market	Mean
Benchmark Model (BM)	4.08	3.85	5.42	16.19	4.36
Scenario A	4.03	4.00	5.51	13.95	4.34
Scenario B	4.02	3.96	5.53	14.27	4.33
Relative deviation of BM-A	−1.16%	3.84%	1.61%	−13.82%	−0.67%
Relative deviation of A-B	−0.39%	−1.01%	0.34%	2.28%	−0.31%
**Trading Volume**	**SHM Market**	**SZM Market**	**SB Market**	**STAR Market**	**Mean**
Benchmark Model (BM)	728,746	500,856	187,637	37,503	1,454,742
Scenario A	621,147	436,424	187,990	32,352	1,277,914
Scenario B	619,974	433,993	189,696	31,683	1,275,346
Relative deviation of BM-A	−14.76%	−12.86%	0.19%	−13.73%	−12.16%
Relative deviation of A-B	−0.19%	−0.56%	0.91%	−2.07%	−0.20%

**Table 6:** Market pricing efficiency results for scenarios A and B, and BM model.

MAE	SHM Market	SZM Market	SB Market	STAR Market	Mean
Benchmark Model (BM)	0.532	0.528	0.980	6.103	2.035
Scenario A	0.287	0.431	0.933	4.995	1.662
Scenario B	0.267	0.379	0.993	4.989	1.657
Relative deviation of BM-A	−45.96% **(0.000)**	−18.27% **(0.007)**	−4.81% (0.309)	−18.15% **(0.012)**	−18.37% **(0.002)**
Relative deviation of A-B	−7.05% **(0.000)**	−12.04% **(0.000)**	6.51% (0.443)	−0.13% **(0.013)**	−0.27% **(0.002)**
**MRE**	**SHM Market**	**SZM Market**	**SB Market**	**STAR Market**	**Mean**
Benchmark Model (BM)	5.39	6.43	8.33	20.79	10.23
Scenario A	2.84	4.42	7.03	17.28	7.89
Scenario B	2.64	3.91	7.66	16.48	7.68
Relative deviation of BM-A	−47.26% **(0.000)**	−31.28% **(0.003)**	−15.51% (0.107)	−16.89% **(0.000)**	−22.87% **(0.000)**
Relative deviation of A-B	−7.03% **(0.000)**	−11.49% **(0.000)**	8.97% (0.270)	−4.60% **(0.000)**	−2.76% **(0.000)**

**Table 7:** Investor return results for scenarios A and B, and BM model.

Annualized Return Rate	Institution	XLinvestor	Linvestor	Minvestor	Sinvestor	Retailer
Benchmark Model (BM)	−2.90%	−9.55%	−16.95%	6.46%	6.92%	9.29%
Scenario A	2.14%	−8.50%	−14.65%	−2.32%	−7.89%	2.45%
Scenario B	−0.99%	−5.74%	−11.81%	−3.63%	−4.93%	4.17%
Relative deviation of BM-A	5.04%	1.06%	2.30%	−8.78%	−14.81%	−6.84%
Relative deviation of A-B	−3.13%	2.76%	2.84%	−1.31%	2.96%	1.73%
**Max Drawdown**	**Institution**	**XLinvestor**	**Linvestor**	**Minvestor**	**Sinvestor**	**Retailer**
Benchmark Model (BM)	2.32%	6.30%	8.09%	6.38%	8.68%	8.58%
Scenario A	1.24%	2.68%	4.98%	4.15%	4.57%	3.27%
Scenario B	1.56%	2.09%	3.62%	4.19%	4.54%	3.08%
Relative deviation of BM-A	−46.45%	−57.44%	−38.49%	−35.06%	−47.41%	−61.87%
Relative deviation of A-B	25.28%	−21.96%	−27.30%	1.00%	−0.65%	−5.72%

**Table 8:** Market volatility results for scenarios C and D, and BM model.

Volatility of Transaction Price	SHM Market	SZM Market	SB Market	STAR Market	Mean
Benchmark Model (BM)	66.0	73.6	77.0	84.9	75.4
Scenario C	67.1	70.1	78.8	83.4	74.8
Scenario D	68.4	75.1	79.8	85.2	77.1
Relative deviation of BM-C	1.73%	−4.71%	2.24%	−1.87%	−0.72%
Relative deviation of BM-D	3.75%	2.09%	3.52%	0.30%	2.32%
**Volatility of Mid-Point of Bid and Ask Prices**	**SHM Market**	**SZM Market**	**SB Market**	**STAR Market**	**Mean**
Benchmark Model (BM)	57.4	67.2	70.6	92.0	71.8
Scenario C	58.7	63.1	72.5	89.4	70.9
Scenario D	60.4	69.0	74.0	93.0	74.1
Relative deviation of BM-C	2.29%	−6.03%	2.60%	−2.84%	−1.22%
Relative deviation of BM-D	5.23%	2.68%	4.71%	1.06%	3.17%

**Table 9:** Market liquidity results for scenarios C and D, and BM model.

Bid–Ask Spread	SHM Market	SZM Market	SB Market	STAR Market	Mean
Benchmark Model (BM)	4.08	3.85	5.42	16.19	4.36
Scenario C	4.00	4.14	5.80	15.37	4.41
Scenario D	3.97	3.82	5.19	15.10	4.24
Relative deviation of BM-C	−1.96%	7.55%	6.93%	−5.09%	−2.70%
Relative deviation of BM-D	−2.70%	−0.80%	−4.32%	−6.71%	−3.92%
**Trading Volume**	**SHM Market**	**SZM Market**	**SB Market**	**STAR Market**	**Mean**
Benchmark Model (BM)	728,746	500,856	187,637	37,503	1,454,742
Scenario C	707,800	458,730	176,511	36,883	1,379,923
Scenario D	744,284	515,251	190,363	36,345	1,486,243
Relative deviation of BM-C	−2.87%	−8.41%	−5.93%	−1.65%	−5.14%
Relative deviation of BM-D	2.13%	2.87%	1.45%	−3.09%	2.17%

**Table 10:** Market pricing efficiency results for scenarios C and D, and BM model.

MAE	SHM Market	SZM Market	SB Market	STAR Market	Mean
Benchmark Model (BM)	0.532	0.528	0.980	6.103	2.0
Scenario C	0.482	0.552	1.056	5.860	2.0
Scenario D	0.468	0.570	0.938	5.765	1.935
Relative deviation of BM-C	−9.29% (0.212)	4.48% (0.354)	7.83% (0.230)	−3.98% (0.447)	−2.35% (0.457)
Relative deviation of BM-D	−11.99% (0.157)	7.93% (0.232)	−4.30% (0.343)	−5.53% (0.416)	−4.93% (0.398)
**MRE**	**SHM Market**	**SZM Market**	**SB Market**	**STAR Market**	**Mean**
Benchmark Model (BM)	5.39	6.43	8.33	20.79	10.2
Scenario C	5.01	5.73	8.27	19.61	9.7
Scenario D	5.15	6.77	8.18	19.97	10.02
Relative deviation of BM-C	−7.12% (0.291)	−10.83% (0.203)	−0.72% (0.479)	−5.69% (0.241)	−5.68% (0.152)
Relative deviation of BM-D	−4.55% (0.345)	5.28% (0.326)	−1.70% (0.452)	−3.94% (0.242)	−2.11% (0.323)

**Table 11:** Investor return results for scenarios C and D, and BM model.

Annualized Return Rate	Institution	XLinvestor	Linvestor	Minvestor	Sinvestor	Retailer
Benchmark Model (BM)	−2.90%	−29.55%	−16.95%	6.46%	6.92%	9.29%
Scenario C	2.76%	−19.82%	−3.33%	19.02%	13.83%	3.29%
Scenario D	−20.53%	−43.81%	−18.04%	0.08%	11.94%	25.63%
Relative deviation of BM-C	5.66%	9.74%	13.62%	12.57%	6.91%	−6.00%
Relative deviation of BM-D	−17.63%	−14.26%	−1.10%	−6.38%	5.03%	16.34%
**Max Drawdown**	**Institution**	**XLinvestor**	**Linvestor**	**Minvestor**	**Sinvestor**	**Retailer**
Benchmark Model (BM)	2.32%	6.30%	8.09%	6.38%	8.68%	8.58%
Scenario C	2.08%	5.99%	5.17%	5.13%	5.37%	6.58%
Scenario D	4.47%	7.93%	7.94%	7.02%	7.58%	7.83%
Relative deviation of BM-C	−10.35%	−4.97%	−36.13%	−19.58%	−38.19%	−23.24%
Relative deviation of BM-D	92.35%	25.91%	−1.89%	9.96%	−12.63%	−8.72%

**Figure A1:**
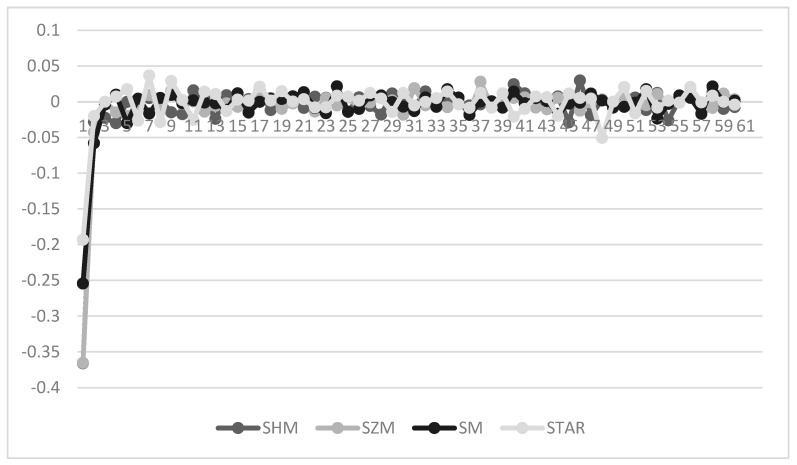
Autocorrelation coefficients of the returns of four assets.

**Figure A2:**
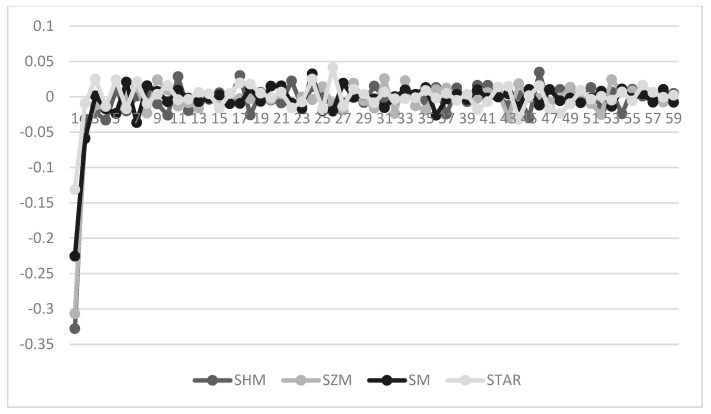
Autocorrelation coefficients of the midpoint price returns of four assets.

**Figure A3:**
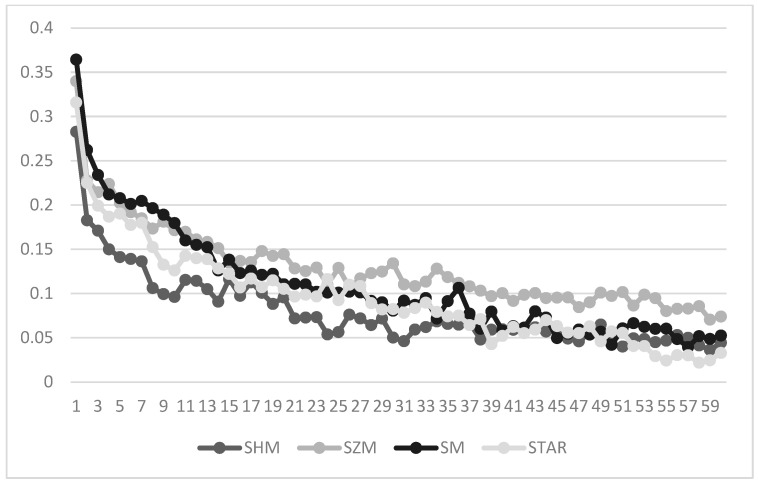
Autocorrelation coefficients of absolute returns for four assets.

**Figure A4:**
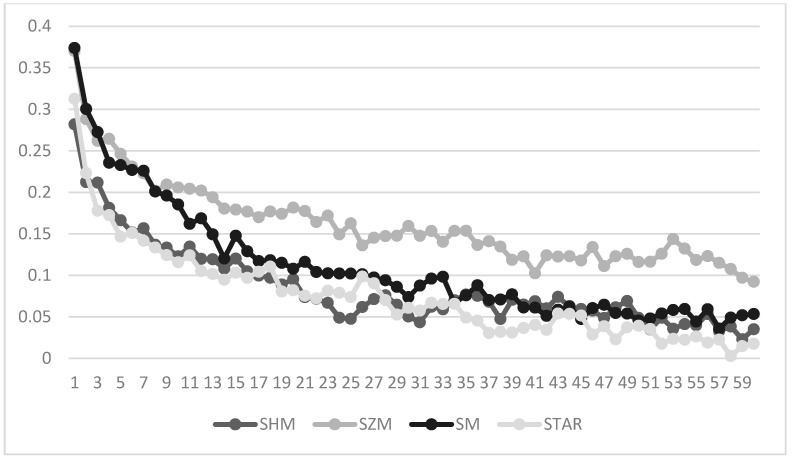
Autocorrelation coefficients of absolute returns of the four assets’ midpoint prices.

## Data Availability

The data presented in this study are available on request from the corresponding author.

## Appendix A. Model Calibration

To demonstrate that the proposed model can effectively simulate the characteristics of real markets, we conducted model calibration on the formatted features of the real market, such as excess kurtosis fat tails and volatility clustering. The specific results are shown below.

Firstly, the distribution of price returns in real markets has a characteristic of excess kurtosis fat tails, which was also exhibited in our simulation model. As shown in Table A1, the price returns in our simulation model exhibited the characteristic of excess kurtosis fat tails, where the minute returns of the four assets (Shanghai main-board (SHM), Shenzhen main-board (SZM), Second-board (SB), and Sci-Tech Innovation board (STAR)) all had a kurtosis significantly greater than 3, while their skewness was not equal to zero.

**Table A1:** Basic statistical characteristics of the returns of each asset.

Market	Skewness	Kurtosis
SHM	−0.0749	7.1592
SZM	−0.1238	9.347
SB	0.2215	21.5579
STAR	0.2302	22.6829

Secondly, empirical studies have found no autocorrelation in stock returns and midpoint returns in real markets. The four assets in our model also exhibited this feature, as shown in Figure A1 and Figure A2, where the autocorrelation coefficients of the asset returns and midpoint returns were negative for a very short period of time, but then rapidly decayed to zero, consistent with the lack of autocorrelation in real markets.

Thirdly, volatility clustering is observed in real markets, and our model replicated this feature well through the characteristics of autocorrelation coefficients of the squares of asset returns and midpoint returns. As shown in Figure A3 and Figure A4, both sets of autocorrelation coefficients exhibited slow decay, indicating that the absolute value of asset returns and the square of midpoint returns have long memory and are consistent with the volatility clustering in real markets.
